# Molecular analysis of blood-associated pathogens in common ravens (*Corvus corax*) from Germany

**DOI:** 10.1007/s00436-025-08510-3

**Published:** 2025-05-31

**Authors:** Aline Lamien-Meda, Josef Harl, Astrid Lieber, Maria Sophia Unterköfler, Barbara Eigner, Licha N. Wortha, Franz Müller, Mike Heddergott, Hans-Peter Fuehrer

**Affiliations:** 1 Institute of Parasitology, Department of Biological Sciences and Pathobiology, University of Veterinary Medicine Vienna, Veterinaerplatz 1, 1210 Vienna, Austria; 2 Clinical Institute of Pathology, Medical University Vienna, Waehringer Guertel 18-20, 1090 Vienna, Austria; 3 Wildlife Biology Working Group, Justus-Liebig-Universität Gießen, Leihgesterer Weg 217, 35392 Giessen, Germany; 4 Department of Zoology, Musée National d’Histoire Naturelle, 25, Rue Muenster, 2160 Luxembourg, Luxembourg

**Keywords:** *Corvus corax*, Filarioid nematodes, *Trypanosoma*, Haemosporidian parasites, *Leucocytozoon*, *Haemoproteus*

## Abstract

Common ravens (*Corvus corax*) are intelligent scavengers that adapt to diverse environments, playing a key ecological role, but their health and ecosystem contributions can be affected by parasites. This study investigates the prevalence and diversity of blood parasite infections in common ravens using molecular techniques. Blood samples (*n* = 42) were collected from dead common ravens in Germany and screened for filarioid nematodes, trypanosomatids, and haemosporidian parasites. The results showed that 26.2% of the common ravens were PCR-positive for at least one parasite, with some cases of mixed infections. Filarioid nematodes were found in 16.7%, trypanosomatids in 4.8%, and haemosporidian parasites in 16.7% of the common ravens. Sequencing revealed the presence of four *Leucocytozoon CytB* lineages and one *Haemoproteus* lineage. The findings suggest that common ravens in Germany are often infected with diverse avian blood parasites, with a higher prevalence of filarioid nematodes. Further research is needed to confirm the circulation of these parasites in the common raven population and to identify the specific filarioid nematode species present in Germany.

## Introduction

Blood parasites from the genera *Plasmodium*, *Haemoproteus*, and *Leucocytozoon*, which are transmitted by dipteran vectors such as mosquitoes, biting midges, and blackflies, are widespread among bird populations and can cause mild to severe infections and life-threatening diseases. Studying blood parasites in wild bird populations is important for understanding host-parasite co-evolution, the ecological role of parasitism, and the impact of environmental factors on transmission dynamics (Valkiunas 2004).

Common ravens (*Corvus corax*), known for their intelligence, adaptability, and wide distribution, are an intriguing species for studying blood parasite infections. As large, long-living scavengers, these ravens inhabit a variety of ecosystems, ranging from urban environments to remote wilderness areas, exposing them to diverse environmental pressures and parasite transmission risks. Social behavior, such as communal roosting and group foraging, may also increase the likelihood of parasite exposure and transmission (Boarman and Heinrich 1999). Despite their ecological significance, there is limited research on blood parasite prevalence and diversity in wild raven populations, with most parasite studies of birds focusing on smaller songbirds or captive populations (Shurulinkov et al. 2018).

Previous studies of avian blood parasites have shown variable infection rates, influenced by factors such as habitat, climate, and host characteristics such as age, sex, and immune status (Atkinson and Van Atkinson 1991; Hamer et al. 2013). Parasite prevalence and intensity often peak during the breeding season when birds experience increased stress and physiological demands (Marzal et al. 2005). Understanding how these factors impact raven populations is critical as parasitism could affect their fitness, reproductive success, and overall survival.

Recent studies highlight corvids as important hosts for haemosporidian parasites, particularly *Leucocytozoon* and *Plasmodium*. In Bulgaria, wild common ravens showed a 49% prevalence of *Plasmodium* and 31% of *Leucocytozoon*, with some *Leucocytozoon* lineages being raven-specific (Shurulinkov et al. 2018). In southwest Germany, *Leucocytozoon* was the most prevalent genus, infecting 85.3% of carrion crows (*Corvus corone*) and all sampled Eurasian magpies (*Pica pica*), with 65.3% of samples showing multiple infections (Schmid et al. 2017). These findings underscore the high diversity and prevalence of haemosporidian parasites in corvids. In Germany, no specific studies focusing on common ravens’ blood parasites were identified in the literature.

In this study we investigated the prevalence and diversity of blood parasite infections in common ravens from Germany. Using molecular diagnostic techniques, we evaluated the presence of filarioid nematodes, and members of the genera *Trypanosoma*, *Plasmodium*, *Haemoproteus*, and *Leucocytozoon* in 42 blood samples of common ravens collected during autopsy.

## Material and methods

**Fig. 1:**
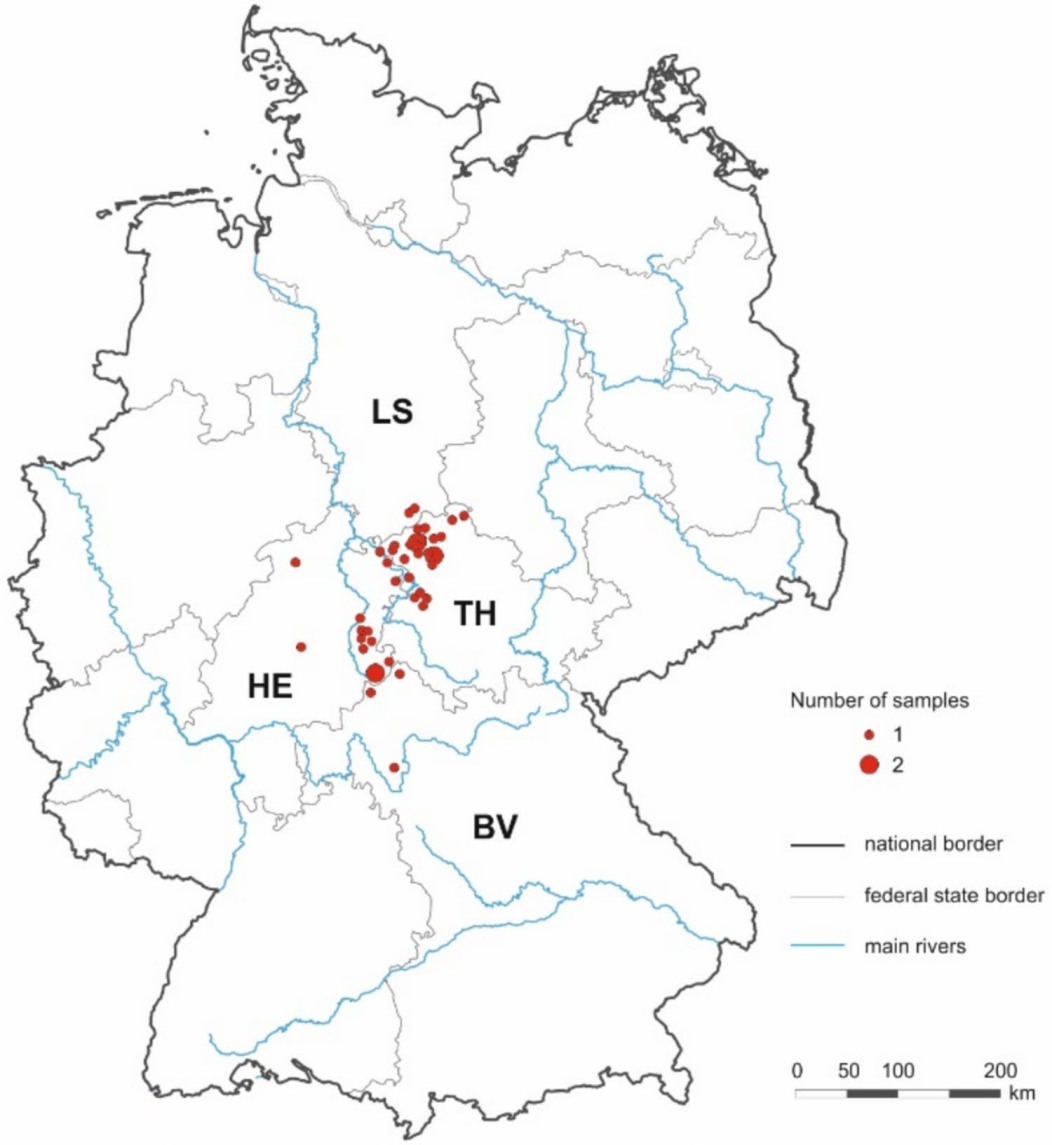
Sampling locations in German federal states: Thuringia (TH), Hessen (HE), Bavaria (BV), and Lower Saxony (LS). The red dots 1 and 2 represent the number of samples per location; black lines represent the national border, grey lines the federal state border, and blue lines the main rivers. The graphic was created with CorelDRAW 2021 (Corel, Ottawa, Canada)

A total of 42 blood samples were collected from dead common ravens (15 male and 27 females) during autopsy. The birds were collected between September 2003 and August 2016 in four federal states (Thuringia, Hesse, Bavaria, and Lower Saxony) of Germany (Fig. 1). The location, date, sex, and growth stage (immature or adult) of the birds were recorded whenever possible. The age was determined using the usual throat coloration for *Corvus* species (Kalchreuter 1971). According to this, immature common ravens (< 2 years) have a red coloration and adult birds (> 2 years) have a black coloration. From each blood sample, DNA was extracted using the DNeasy Blood and Tissue Kit (QIAGEN, Hilden, Germany) according to the manufacturer’s instructions, and used to screen for DNA of filarioid nematodes, *trypanosomatids*, *Plasmodium*, *Haemoproteus*, and *Leucocytozoon* parasites.

All PCRs were performed in 25 µl volumes with the GoTaq G2 Flexi DNA Polymerase kit (Promega GmbH, Walldorf, Germany). The mastermix contained 14.375 µl nuclease-free water, 5 µl 5X Green Reaction Buffer, 2 µl MgCl_2_ (25 mM), 0.5 µl dNTPs (10 mM), 0.125 µl GoTaq G2 Polymerase, each 1 µl each primer (10 mM), and 1–4 µl DNA template. The PCRs were run under the following conditions: initial denaturation for 2 min at 94 °C, followed by 35 cycles with 30 s at 94 °C, 30 s at X °C (see annealing temperatures provided for primers), 1 min at 72 °C, and a final extension for 10 min at 72 °C.

To detect filarioid nematodes, we used two separate primer sets targeting partial sequences of the nuclear 18S rRNA gene (18S) and the mitochondrial cytochrome c oxidase subunit I (*COI*). The primers ChandFO (5′-GAG ACC GTT CTC TTT GAG GCC-3′) and ChandRO (5′-GTC AAG GCG TAN NTT TAC CGC CGA-3′) (Hamer et al. 2013) target a 550 bp section of the 18S, and the primers COIintF (5′-TGA TTG GTG GTT TTG GTA A-3′) and COIintR (5′-ATA AGT ACG AGT ATC AAT ATC-3′) (Casiraghi et al. 2001; Merkel et al. 2007) target a 649 bp section of the *COI*. The annealing temperatures for the two primer sets were 57 °C (18S) and 51 °C (*COI*).

To detect *Trypanosoma* parasites, we used a nested PCR targeting an 870 bp section of the 18S as previously described (Peña-Espinoza et al. 2023) with the nest 1 primers Tryp_18S_F1 (5′-GTG GAC TGC CAT GGC GTT GA-3′) and Tryp_18S_R1 (5′-CAG CTT GGA TCT CGT CCG TT GA-3′) and the nest 2 primers Tryp_18S_F2 (5′-CGA TGA GGC AGC GAA AAG AAA TAG AG-3′) and Tryp_18S_R2 (5′-GAC TGT AAC CTC AAA GCT TTC GCG-3′). The annealing temperature used in the PCRs was 56 °C.

To detect haemosporidian parasites belonging to the genera *Plasmodium*, *Haemoproteus*, and *Leucocytozoon*, we used the common nested PCR assay (Hellgren et al. 2004), which targets a 476 bp section of the mitochondrial cytochrome b (*CytB*) gene. The first PCR was performed with the primers HaemNFI (5′-CAT ATA TTA AGA GAA NTA TGG AG-3′) and HaemNR3 (5′-ATA GAA AGA TAA GAA ATA CCA TTC-3′). The nested PCRs were performed with the primers HaemF (5′-ATG GTG CTT TCG ATA TAT GCA TG-3′) and HaemR2 (5′-GCA TTA TCT GGA TGT GAT AAT GGT-3) targeting *Plasmodium* and *Haemoproteus* parasites, and HaemFL (5′-ATG GTG TTT TAG ATA CTT ACA TT-3′) and HaemR2L (5′-CAT TAT CTG GAT GAG ATA ATG GNG C-3′) targeting *Leucocytozoon* parasites (Bensch et al. 2000; Hellgren et al. 2004). For the nested PCRs, each 1 µl of PCR product from the first PCR was used as template. The annealing temperature used for the PCRs with all three primer sets was 48 °C.

The PCR products were visualized on 1.8% agarose gels stained with Midori Green Advance (Biozym Scientific GmbH, Oldendorf, Germany) under UV light. PCR-positive samples were sent to LGC Genomics GmbH (Berlin, Germany) for purification and sequencing (using the PCR primers). The sequences were assembled and visually inspected using BioEdit v.7.7.1 (Hall 1999). For the phylogenetic analysis, additional nucleotide sequences were retrieved from NCBI GenBank using BLAST search and aligned and sorted with MAFFT v.7 (Katoh and Standley 2013) using the default options. Genetic distances were calculated with MEGA X v.10.0.5 (Kumar et al. 2018).

To infer the phylogenetic position of the *Trypanosoma* parasite lineage obtained in the present study, one of the 18S sequences was subjected to BLAST search on NCBI GenBank, and all sequences covering the entire 884 bp sequence section and showing a genetic similarity between 90 and 100% were downloaded. The resulting 336 hits from GenBank and the two sequences obtained in the present study were aligned and sorted with MAFFT v.7 (Katoh and Standley 2013) using the default options. The 338 sequences were aligned, and a *m*aximum *l*ikelihood (ML) bootstrap tree (1000 replicates, model GTR + G + I) was calculated with IQ-TREE v.2.3.6 (Nguyen et al. 2015). Based on the latter tree (not shown), we identified the clade containing the *Trypanosoma* sequences obtained in the present study. Using the 21 sequences in the latter clade and a sequence of *Trypanosoma melophagium* (ON637628) as the outgroup, we calculated ML and Bayesian Inference (BI) trees. The alignment contained 903 positions, of which 32 positions contained gaps and were excluded before the analyses. The best-fit substitution model based on the corrected Akaike information criterion (cAIC) was evaluated with IQ-TREE v.2.3.6 (Nguyen et al. 2015), resulting in the model TIM3e + I + G4. Since the latter model is not implemented in MrBayes v.3.2.7 (Ronquist et al. 2012), the next similar model GTR + G + I was used for the phylogenetic analyses. The BI tree was calculated with MrBayes v.3.2.7 (Ronquist et al. 2012) by running 5 million generations and sampling every thousandth tree; the first 25% of trees were discarded as burn-in, and a 50% majority rule consensus tree was calculated from the remaining 3750 trees. An ML majority-rule consensus tree (1000 bootstrap replications) was calculated with IQ-TREE v.2.3.6 (Nguyen et al. 2015).

A similar approach was followed to determine the phylogenetic position of the Filarioidea identified in the present study based on partial 649 bp *COI* sequences. The *COI* sequence was subjected to BLAST search and all hits covering at least 90% of the sequence were downloaded. The sequences were aligned and sorted with MAFFT v.7 (Katoh and Standley 2013) using the default options. After removing all sequences with ambiguities, the alignment contained 991 sequences. An ML bootstrap tree (1000 replicates, model GTR + G + I) was calculated with IQ-TREE v.2.3.6 (Nguyen et al. 2015). Based on the latter tree (not shown), we identified a clade with maximum support containing the *Filarioidea* sequences obtained in the present study. Using the 34 sequences from the latter clade and a sequence of *Dirofilaria repens* (OP494253) as the outgroup, we calculated BI and ML trees applying the same settings as for the *Trypanosoma* 18S tree, using the model GTR + G + I as suggested by IQ-TREE v.2.3.6 (Nguyen et al. 2015).

For the phylogenetic tree based on partial 476 bp *CytB* sequences of *Leucocytozoon* parasites, we followed a different approach. Since more than 1000 different *CytB* lineages of avian *Leucocytozoon* parasites have been identified, we only included *Leucocytozoon* lineages found in *Corvus* species. The alignment included 50 unique lineages from seven different *Corvus* species and a sequence of *Leucocytozoon toddi* BUBT2 (OL598451) as the outgroup. The BI and ML trees were calculated using the same settings as the *Trypanosoma* and Filarioidea trees, using the model GTR + G + I as suggested by IQ-TREE v.2.3.6 (Nguyen et al. 2015).

All trees were visualized with FigTree v.1.4.3 (http://tree.bio.ed.ac.uk/software/figtree/) and graphically prepared with Adobe Illustrator CC v.2015 (Adobe Inc., San Jose, CA, USA).

The 95% confidence interval (CI) for prevalence percentages in the samples was calculated using the binomial proportion method with the following formula:

$${\varvec{C}}{\varvec{I}}={\varvec{p}}\pm {\varvec{Z}}\times \surd \left[{\varvec{p}}\left(1-{\varvec{p}}\right)/{\varvec{n}}\right]$$

where *p* is the observed prevalence (proportion of infected individuals), *Z* is the *Z*-score corresponding to the desired confidence level (e.g., 1.96 for 95% CI), and *n* is the sample size.

## Results

The sample included 42 common ravens (KR1 to KR42) with 38.1% adult (16/42) and 61.9% immature individuals (26/42). The adults had a higher average weight (1206.9 g, 95% confidence interval CI: 1151.3–1262.5 g) compared to immatures (1061.2 g, 95% CI: 1023.4–1099.0 g). 35.7% were males (15/42) and 64.3% were females (27/42), with males showing a slightly higher average weight (1157.3 g, 95% CI: 1094.5–1220.1 g) than females (1110.7 g, 95% CI: 1074.6–1146.8 g).

**Fig. 2:**
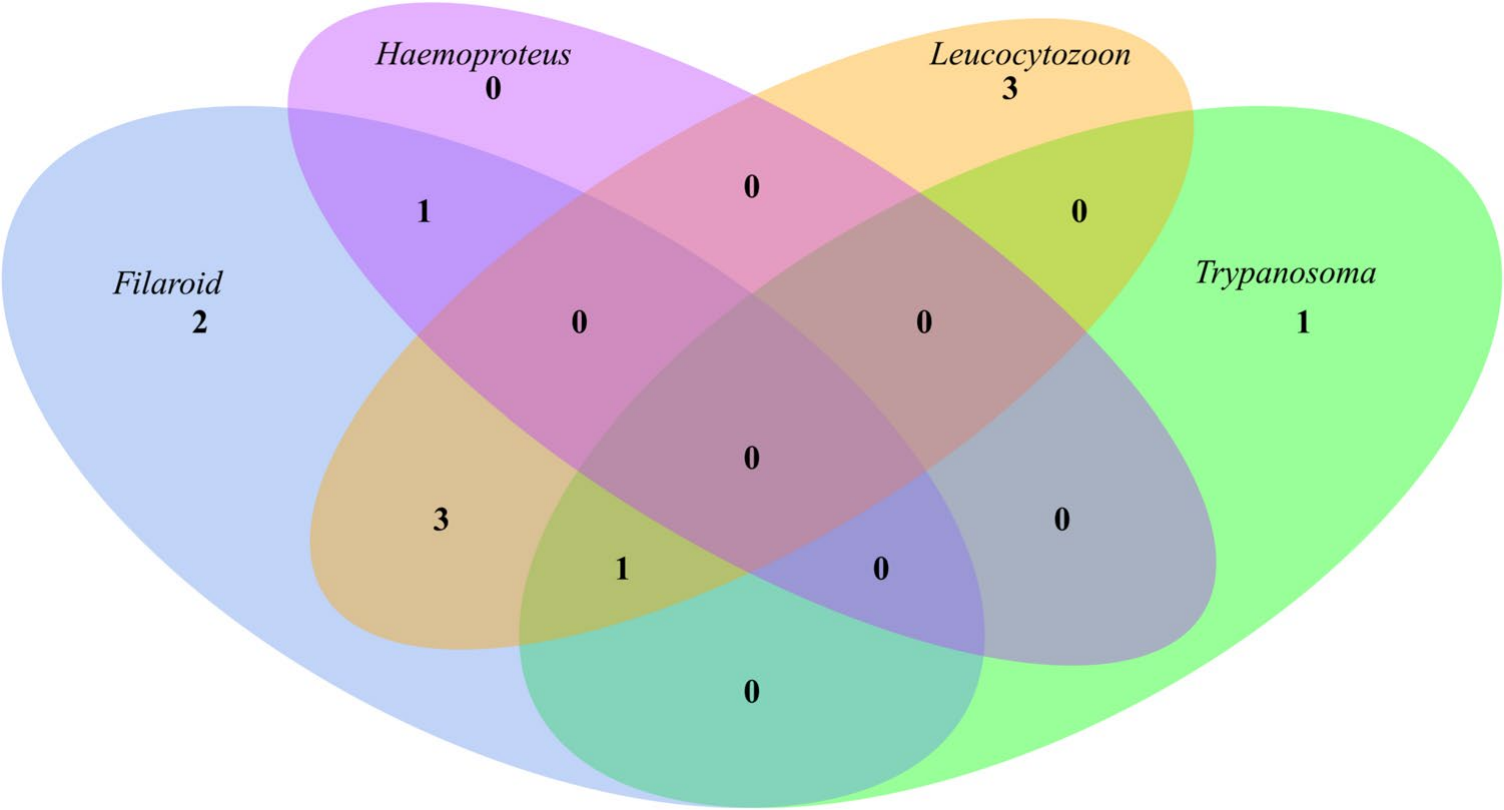
Venn diagram of parasite prevalence in the studied samples. Numbers represent counts of common ravens (*Corvus corax*) with respective pathogens detected (blue for filarioid, green for *Trypanosoma*, yellow for *Leucocytozoon*, and purple for *Haemoproteus*). The overlapping colors and representing co-infections

The PCR screenings and sequencing revealed that altogether 11/42 birds were infected with blood parasites. Filarioid nematodes were present in seven common ravens (16.7%, 95% CI: 5.1–28.3%), *Trypanosoma* sp. in two individuals (4.8%, 95% CI: 0–11.4.3%), *Leucocytozoon* sp. in seven individuals (16.7%, 95% CI: 5.1–28.3%) with one individual harboring a co-infection with 2 lineages (Table 2), and *Haemoproteus* sp. in one individual (2.4%; 95% CI: 0–7.1%) (Tables 1 and 2; Fig. 2). Mixed infections were detected in five (11.9%) individuals, one triple infection with filarioids/*Trypanosoma*/*Leucocytozoon*, one double infection with filarioids/*Haemoproteus*, and three double infections with filarioids/*Leucocytozoon* (Tables 1 and 2; Fig. 2).

**Table 1:** Prevalence and 95% confidence interval (CI) of parasitic infections in the 42 common ravens (*Corvus corax*) using binomial proportion method

Infection type	Number of birds	Prevalence (%)	95% confidence interval (CI)
**Single infections**			
Filarioid nematodes	7	16.7	[5.1%, 28.3%]
*Trypanosoma* sp.	2	4.8	[0%, 11.4%]
*Leucocytozoon* sp.	7	16.7	[5.1%, 28.3%]
*Haemoproteus* sp.	1	2.4	[0%, 7.1%]
**Mixed infections**			
Double infections	4	9.5	[0.7%, 18.3%]
Triple infections	1	2.4	[0%, 7.1%]
**Total infected birds**	11	26.2	[13.1%, 39.3%]

**Table 2:** Positive samples with their GenBank accession numbers and corresponding lineages and parasites

*Sample no*	GenBank no.^*^	*Parasite*	*Lineage*	*Closest BLAST hit*
*KR20*	PV236021	*Leucocytozoon* sp.	EUSE1	JX507218 (100%)
*KR24*	PV236024	*Leucocytozoon* sp.	CCORAX03	MG209764 (100%)
*KR25*	PV226108	*Trypanosoma* sp.	ANI54	JN006849 (100%)
*KR26*	PV243878 PV236148 PV236023 PV236028 PV226109	*Filarioidea* sp. *Filarioidea* sp. *Leucocytozoon* sp. *Leucocytozoon* sp. *Trypanosoma* sp.	MI1 GLH-2012 911E_MF COCOR03 CCORAX03 ANI54	JQ867040 (100%) MT800770 (92.64%) JX867112 (100%) MG209764 (100%) JN006849 (100%)
*KR30*	PV243879 PV236149	*Filarioidea* sp. *Filarioidea* sp.	MI1 GLH-2012 911E_MF	JQ867040 (100%) MT800770 (92.64%)
*KR31*	PV236025	*Leucocytozoon* sp.	CCORAX03	MG209764 (100%)
*KR32*	PV243880 PV236149 PV236020	*Filarioidea* sp. *Filarioidea* sp. *Haemoproteus* sp.	MI1 GLH-2012 911E_MF CXPIP27/FES-2014/H360	JQ867040 (100%) MT800770 (92.64%) KJ579154/KJ128983/KJ488905 (100%)
*KR36*	PV243881	*Filarioidea* sp.	MI1 GLH-2012	JQ867040 (100%)
*KR38*	PV243882 PV236026	*Filarioidea* sp. *Leucocytozoon* sp.	MI1 GLH-2012 CCORAX03	JQ867040 (100%) MG209764 (100%)
*KR39*	PV243883 PV236022	*Filarioidea* sp. *Leucocytozoon* sp.	MI1 GLH-2012 COCOR13	JQ867040 (100%) KJ128991 (100%)
*KR40*	PV243884 PV236027	*Filarioidea* sp. *Leucocytozoon* sp.	MI1 GLH-2012 CCORAX03	JQ867040 (100%) MG209764 (100%)

^*^GenBank accession numbers of sequences from the present study

## Filarioid nematodes

**Fig. 3:**
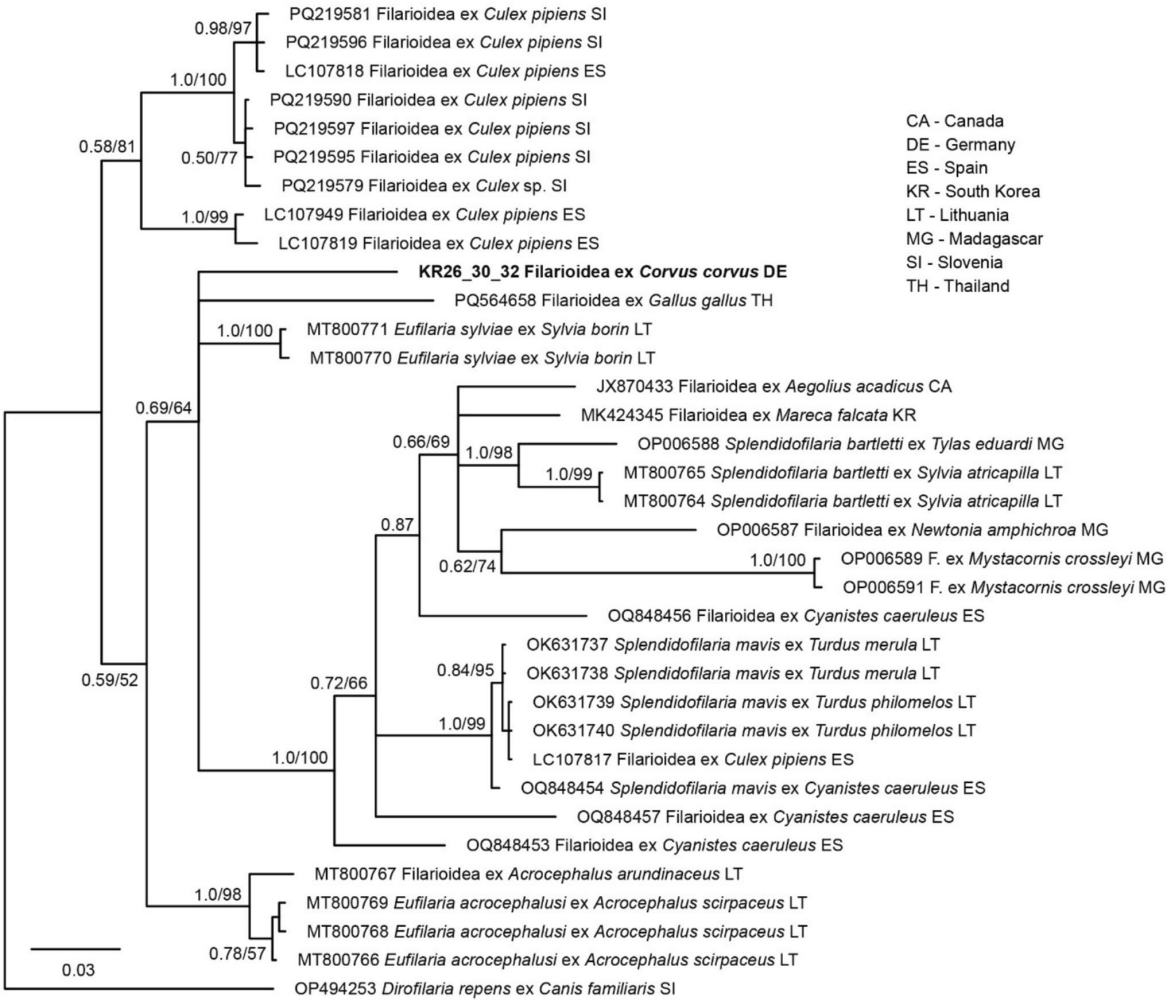
Bayesian Inference tree of closely related Filarioidea parasites based on partial *COI* sequences (649 positions) from the present study and NCBI GenBank. Bayesian posterior probabilities and maximum likelihood bootstrap values are indicated at most nodes. The scale bar indicates the expected mean number of substitutions per site according to the model of sequence evolution applied. The sequences obtained in the present study are marked in bold letters

Filarioid nematodes were found in seven (16.7%) common ravens (KR26, KR30, KR32, KR36, KR38, KR39, KR40) using the PCR targeting the 18S (Table 2). The 18S sequences (PV243878–PV243884) were all identical in the 547 bp section analyzed and 100% matched a Filarioidea sequence (JQ867040) isolated from *Quiscalus quiscula* in the USA. The next-best matches were Filarioidea sequences isolated from Eurasian blue tit (*Cyanistes caeruleus*) in Spain (OQ859189), a dog in the USA (MH390715) (Boyd et al. 2019), and American robin (*Turdus migratorius*) in the USA (JQ867026), with 99.82%, 99.62%, and 99.58% similarity, respectively. The *COI* sequences (PV236148–PV236150, *Filarioidea* sp.) obtained from three individuals (KR26, KR30, KR32) were identical. The closest matches to our *Filarioidea* sp. were *Eufilaria sylviae* from garden warbler (*Sylvia borin*) (MT800771, MT800770) with 92.64% and 92.43% similarity, and *Eufilaria acrocephalusi* from common reed warbler (*Acrocephalus scirpaceus*) (MT800769, MT800766) with 91.50% similarity, all of which were found in Lithuania. In the phylogenetic tree (Fig. 3), the *COI* lineage formed a single branch in a weakly supported clade (pp = 0.69/bs = 64), which contained Filarioidea lineages from various bird species assigned either to *Eufilaria* or *Splendidofilaria*.

### *Trypanosoma* parasites

**Fig. 4:**
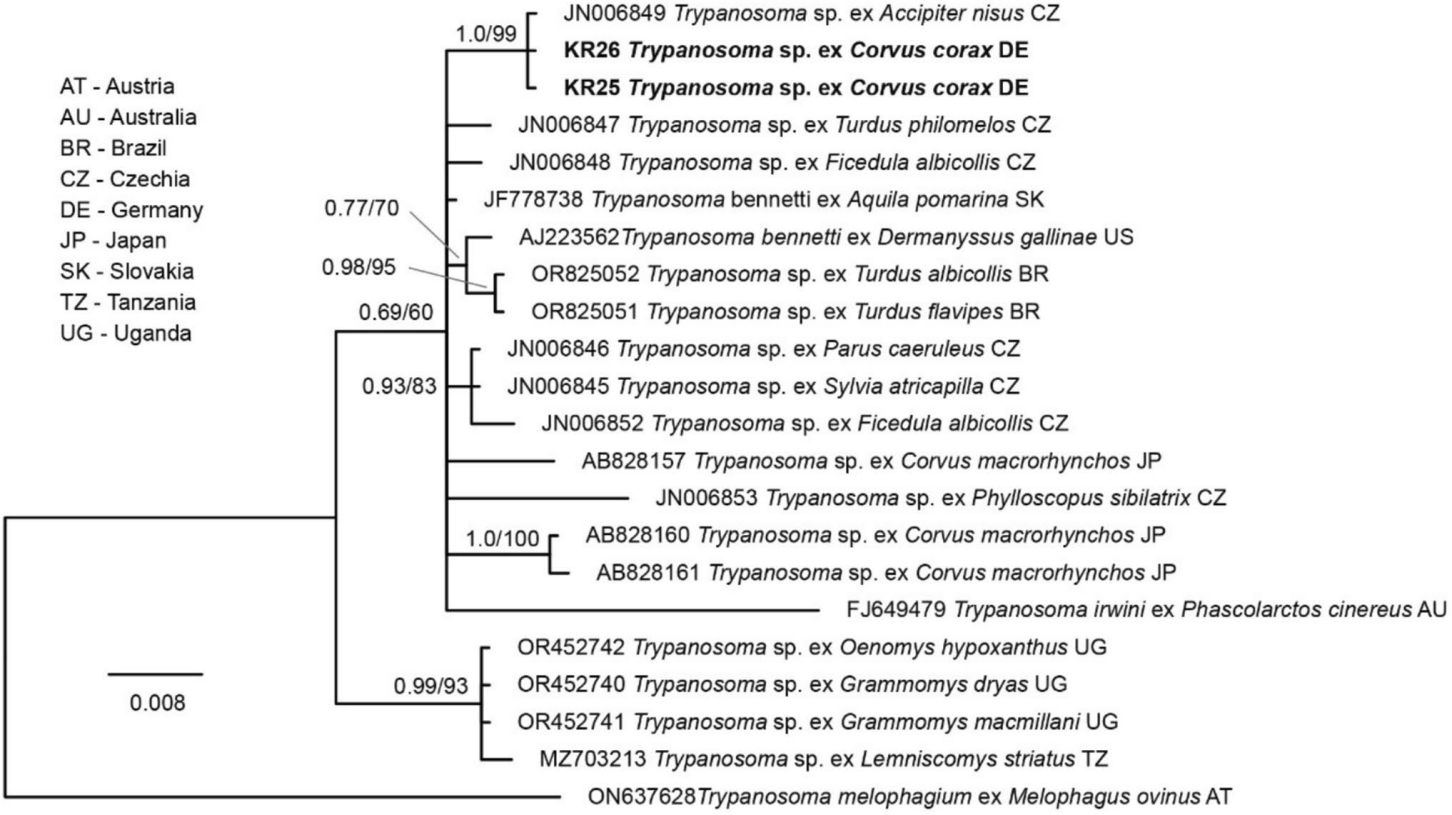
Bayesian Inference tree of closely related *Trypanosoma* parasites based on partial 18S sequences (871 positions excluding gaps) from the present study and NCBI GenBank. Bayesian posterior probabilities and maximum likelihood bootstrap values are indicated at all nodes. The scale bar indicates the expected mean number of substitutions per site according to the model of sequence evolution applied. The sequences obtained in the present study are marked in bold letters

*Trypanosoma* parasites were detected in two common ravens (KR25, KR26) (Table 2). The 18S sequences (PV226108–PV226109) were identical in the 884 bp section analyzed to a *Trypanosoma* sequence identified in Eurasian sparrowhawk (*Accipiter nisus*) from Czechia (JN006849). In the phylogenetic tree (Fig. 4), the lineage clustered together in a weakly supported clade (pp = 0.69/bs = 60) with *Trypanosoma* sequences found in various bird species, but the relationships between the lineages in the latter clade were mostly not resolved, presenting as a polytomy.

## Haemosporidian parasites

**Fig. 5:**
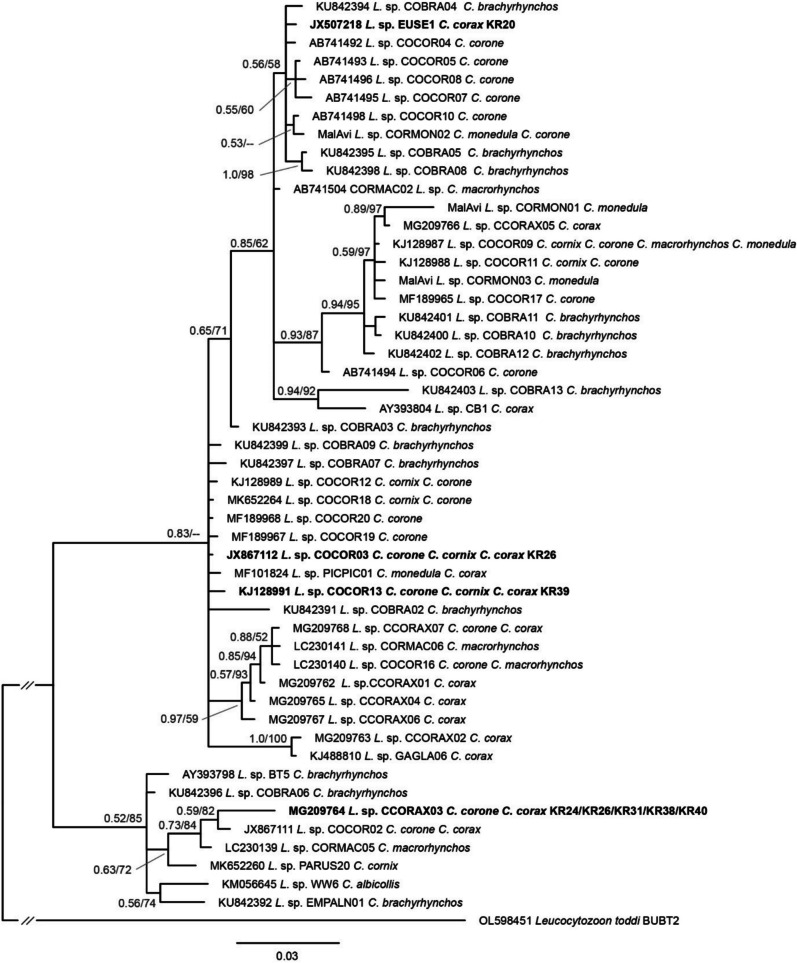
Bayesian Inference tree based on partial (476 bp) *cytb* sequences of *Leucocytozoon* parasites found in birds of the genus *Corvus*. A sequence of *Leucocytozoon toddi* (OL598451) was included as an outgroup. Bayesian posterior probabilities and maximum likelihood bootstrap values are indicated at all nodes. The scale bar indicates the expected number of substitutions per site according to the model of sequence evolution applied. The sequences obtained in the present study are marked in bold letters

*Leucocytozoon* parasites were found in seven (16.7%) common ravens (KR20, KR24, KR26, KR31, KR38, KR39, KR40), with one individual presenting a mixed infection (PV236021–PV236028). The *Leucocytozoon CytB* lineages EUSE1 (JX507218), COCOR03 (JX867112), and COCOR13 (KJ128991) were found in one individual each, and CCORAX03 (MG209764) was found in five individuals (Table 2; Fig. 5). *Haemoproteus* parasites were found in one (2.4%) common raven (KR32; PV236020); the lineage CXPIP27 was previously isolated from *Corvus corone* in Portugal (KJ488905) and *Culex pipiens* in France (KJ579154). None of the haemosporidian parasite lineages has been linked to a morphological species yet. In the phylogenetic tree, the four *Leucocytozoon* lineages cluster together with numerous other lineages that had been found in *C. corax* and other *Corvus* species.

## Discussion

Many studies have investigated blood parasite prevalences in birds, but there are relatively few studies on Corvidae (Rodríguez and Matta 2001; Benedikt et al. 2009; Freund et al. 2016; Scaglione et al. 2016; Schmid et al. 2017). Our study confirmed the presence of trypanosomatids, filarioid nematodes, and haemosporidian parasites in *Corvus corax* by PCR and sequencing.

We found 16.7% of the common ravens to be infected with filarioid nematodes. This prevalence is slightly higher than the prevalence obtained by Hamer et al. (2013) with American robins and house sparrows (1.4–11.1%). Our study is the first to report filarioid nematodes in common ravens. Indeed, the few studies about Corvidae parasites did not investigate the prevalence of filarioid nematodes in these birds (Yoshimura et al. 2014; Freund et al. 2016; Scaglione et al. 2016; Schmid et al. 2017; Shurulinkov et al. 2018). The filarioid 18S rRNA sequences were identical to *Onchocercidae* sp. (JQ867040) from common grackles (*Quiscalus quiscula*) in the USA (Hamer et al. 2013), and the *COI* sequences matched closely with *Eufilaria* or *Splendidofilaria* from various bird species (Binkienė et al. 2021). The detection of the identical 18S rRNA sequence in 16.7% of the samples (2 adult and 5 immature birds) suggests that the detected filarioid circulates in the area studied and might have an impact on the common raven population. As mentioned by Hamer et al. (2013), lice, flies, and biting midges are likely vectors of the filarioid nematodes. Investigating the parasitemia of vectors collected directly on the host could bring more details regarding the source of infection.

The second parasite group with a significant prevalence of 16.7% was *Leucocytozoon*. This prevalence was lower compared to the 31.4% prevalence observed in Bulgarian ravens (Shurulinkov et al. 2018), and much lower compared to prevalences in other *Corvus* species from Germany, Italy, Japan, and the USA (Yoshimura et al. 2014; Freund et al. 2016; Scaglione et al. 2016; Schmid et al. 2017). The *Leucocytozoon* lineage CCORAX03 was found in five individuals; it was previously found in *C. corax* from Bulgaria (Shurulinkov et al. 2018) and *C. corone* in Germany (Strehmann et al. 2023). The lineage COCOR03 (identical to CORMAC03), present in one individual, was previously found in *C. corone* in Germany (Schmid et al. 2017) and hooded crow (*Corvus cornix*) in Italy (Scaglione et al. 2016). The lineage COCOR13, also present in one individual, was found in *C. corone* and *C. cornix* from Austria (Himmel et al. 2019; Harl et al. 2019), *C. corone* from Germany (Schmid et al. 2017), and *C. cornix* from Italy (Scaglione et al. 2016). The fourth lineage, EUSE1, was previously isolated from *Eusimulium securiforme* in Czechia (Synek et al. 2013) and Eurasian jay (*Garrulus glandarius*) from Spain (Illera et al. 2017).

Despite its broad presence, *Leucocytozoon* remains understudied, with significant gaps in understanding its taxonomy, transmission dynamics, and pathogenicity (Valkiūnas 2023). A relatively low prevalence of *Leucocytozoon* in common ravens were observed with a high genetic diversity. These findings highlight a relatively low prevalence of *Leucocytozoon* in common ravens with a genetic diversity of *Leucocytozoon*, emphasizing the need for further molecular studies to understand infection patterns and host-parasite coevolution.

The prevalence of *Haemoproteus* was much lower in the present study (2.7%) than in other studies of corvids (Murata 2002; Scaglione et al. 2016; Schmid et al. 2017). The lineage CXPIP27, found in one individual, was previously detected in *C. cornix* in Italy (Scaglione et al. 2016), *C. corone* in Portugal (Drovetski et al. 2014), and *Culex pipiens* in France (Zélé et al. 2014). This lineage’s presence in 3 species of the genus *Corvus* (*C. Corax*, *C. Cornix*, and *C. Corone*) and in vectors underscores a host adaptation and the importance of vector-host interactions in *Haemoproteus* transmission.

*Trypanosoma* parasites were detected in two common ravens (4.8%), a much lower prevalence compared to 17–18% in another study (Hamer et al. 2013). The Bayesian Inference tree of closely related *Trypanosoma* parasites based on partial 18S rRNA gene sequences revealed a *Trypanosoma* lineage which had previously been detected in *Accipiter nisus* from Czechia (Zídková et al. 2012). The study of Votypka et al. (2004) on the phylogenetic relationship of *Trypanosoma corvi* with other avian trypanosomes has revealed two well-supported monophyletic clades (the “*Trypanosoma avium*” clade and the “*Trypanosoma corvi*” clade) with no significant differences in their 18S rRNA sequences. Our study provides additional molecular data on *Trypanosoma* in common ravens, but it must be noted that *Trypanosoma* parasites are considerably less host-specific with respect to their bird hosts (Zídková et al. 2012).

The present study confirms the presence of filarioid nematodes, *Leucocytozoon*, *Haemoproteus*, and *Trypanosoma* parasites in common ravens (*Corvus corax*) and their circulation within the study area. Molecular evidence of co-infection with blood parasites in common ravens was identified, and we reported, for the first time, the presence of filarioid nemathodes in this specific Corvidae species. Our findings align with previous phylogenetic data, which suggest a variety of *Leucocytozoon* lineages in corvids, distributed across two distinct clades (Freund et al. 2016). Additionally, the results also highlight the genetic diversity and adaptability of *Trypanosoma*, which appears capable of infecting a wide range of avian hosts.

## Conclusion

Overall, our results show that common ravens in Germany are infected with filarioid nematodes, *Trypanosoma*, *Haemoproteus*, and *Leucocytozoon* parasites. Three out of the four co-infected birds harbored filarioids and *Leucocytozoon* sp., and one bird was co-infected with filarioids and *Haemoproteus* sp. However, our study only found a small number of infected ravens within a geographically restricted area and, therefore, cannot provide evidence for the circulation of the parasites we found within the German raven population as a whole. An additional investigation on a larger population of common ravens in the studied areas is required to confirm and morphologically characterize the filarioid nematode species found in the present study.

## Data Availability

No datasets were generated or analysed during the current study.

## References

[CR1] Atkinson CT, Van Atkinson C (1991) Pathogenicity and epizootiology of avian haematozoa: Plasmodium, Leucocytozoon, and Haemoproteus. In: Bird-Parasite Interactions: Ecology, Evolution, and Behaviour, online edn. Oxford University Press

[CR2] Benedikt V, Barus V, Capek M et al (2009) Blood parasites (Haemoproteus and microfilariae) in birds from the Caribbean slope of Costa Rica. Acta Parasitol 54:197–204. 10.2478/s11686-009-0043-1

[CR3] Bensch S, Stjernman M, Hasselquist D et al (2000) Host specificity in avian blood parasites: a study of Plasmodium and Haemoproteus mitochondrial DNA amplified from birds. Proc R Soc b: Biol Sci 267:1583–1589. 10.1098/rspb.2000.1181 10.1098/rspb.2000.1181 PMC1690711 11007335

[CR4] Binkienė R, Chagas CRF, Bernotienė R, Valkiūnas G (2021) Molecular and morphological characterization of three new species of avian Onchocercidae (Nematoda) with emphasis on circulating microfilariae. Parasit Vectors 14. 10.1186/s13071-021-04614-8 10.1186/s13071-021-04614-8 PMC7934436 33673865

[CR5] Boarman WI, Heinrich B (1999) Common Raven (*Corvus corax*). In: Poole A, Gill F (eds) Birds of the World. Cornell Lab of Ornithology, Philadelphia, PA

[CR6] Boyd M, Santoro D, Craft WF et al (2019) Dermatitis caused by autochthonous *Cercopithifilaria bainae* from a dog in Florida, USA: clinical, histological and parasitological diagnosis and treatment. Vet Dermatol 30:68-e20. 10.1111/vde.12701 30474318 10.1111/vde.12701

[CR7] Casiraghi M, Anderson TJC, Bandi C et al (2001) A phylogenetic analysis of filarial nematodes: comparison with the phylogeny of *Wolbachia* endosymbionts. Parasitology 122:93–103. 10.1017/S0031182000007149 11197770 10.1017/s0031182000007149

[CR8] Drovetski S V., Aghayan SA, Mata VA, et al (2014) Does the niche breadth or trade-off hypothesis explain the abundance-occupancy relationship in avian Haemosporidia? Mol Ecol 23. 10.1111/mec.12744 10.1111/mec.12744 24689968

[CR9] Freund D, Wheeler SS, Townsend AK, et al (2016) Genetic sequence data reveals widespread sharing of *Leucocytozoon* lineages in corvids. Parasitol Res 115. 10.1007/s00436-016-5121-3 10.1007/s00436-016-5121-3 27189064

[CR10] Hall TA (1999) BioEdit: a user-friendly biological sequence alignment editor and analysis program for Windows 95/98/NT. Nucleic Acids Symp Ser 41:95–98

[CR11] Hamer GL, Anderson TK, Berry GE, et al (2013) Prevalence of filarioid nematodes and trypanosomes in American robins and house sparrows, Chicago USA. Int J Parasitol Parasites Wildl 2. 10.1016/j.ijppaw.2012.11.005 10.1016/j.ijppaw.2012.11.005 PMC3862512 24533314

[CR12] Harl J, Himmel T, Valkiunas G, Weissenböck H (2019) The nuclear 18S ribosomal DNAs of avian haemosporidian parasites. Malar J 18. 10.1186/s12936-019-2940-6 10.1186/s12936-019-2940-6 PMC6724295 31481072

[CR13] Hellgren O, Waldenström J, Bensch S (2004) A new PCR assay for simultaneous studies of *Leucocytozoon*, *Plasmodium*, and *Haemoproteus* from avian blood. Journal of Parasitology 90. 10.1645/GE-184R1 10.1645/GE-184R1 15357072

[CR14] Himmel T, Harl J, Kübber-Heiss A, et al (2019) Molecular probes for the identification of avian *Haemoproteus* and *Leucocytozoon* parasites in tissue sections by chromogenic in situ hybridization. Parasit Vectors 12. 10.1186/s13071-019-3536-2 10.1186/s13071-019-3536-2 PMC6547609 31159851

[CR15] Illera JC, López G, García-Padilla L, Moreno Á (2017) Factors governing the prevalence and richness of avian haemosporidian communities within and between temperate mountains. PLoS One 12. 10.1371/journal.pone.0184587 10.1371/journal.pone.0184587 PMC5589241 28880919

[CR16] Kalchreuter H (1971) Criteria of age and sex in the Carrion crow (*Corvus c. corone*). Die Vogelwelt 26:106–112

[CR17] Katoh K, Standley DM (2013) MAFFT multiple sequence alignment software version 7: improvements in performance and usability. Mol Biol Evol 30:772–780. 10.1093/molbev/mst010 23329690 10.1093/molbev/mst010 PMC3603318

[CR18] Kumar S, Stecher G, Li M et al (2018) MEGA X: molecular evolutionary genetics analysis across computing platforms. Mol Biol Evol 35:1547–1549. 10.1093/molbev/msy096 29722887 10.1093/molbev/msy096 PMC5967553

[CR19] Marzal A, De Lope F, Navarro C, Møller AP (2005) Malarial parasites decrease reproductive success: an experimental study in a passerine bird. Oecologia 142. 10.1007/s00442-004-1757-2 10.1007/s00442-004-1757-2 15688214

[CR20] Merkel J, Jones HI, Whiteman NK et al (2007) Microfilariae in GaláPagos penguins (*Spheniscus mendiculus*) and flightless cormorants (*Phalacrocorax harrisi*): Genetics, morphology, and prevalence. J Parasitol 93:495–503. 10.1645/GE-1009R.1 17626340 10.1645/GE-1009R.1

[CR21] Murata K (2002) Prevalence of blood parasites in Japanese wild birds. J Vet Med Sci 64:785–790. 10.1292/JVMS.64.785 12399602 10.1292/jvms.64.785

[CR22] Nguyen LT, Schmidt HA, Von Haeseler A, Minh BQ (2015) IQ-TREE: a fast and effective stochastic algorithm for estimating maximum-likelihood phylogenies. Mol Biol Evol 32:268–274. 10.1093/molbev/msu300 25371430 10.1093/molbev/msu300 PMC4271533

[CR23] Peña-Espinoza M, Em D, Shahi-Barogh B, et al (2023) Molecular pathogen screening of louse flies (Diptera: Hippoboscidae) from domestic and wild ruminants in Austria. Parasit Vectors 16. 10.1186/s13071-023-05810-4 10.1186/s13071-023-05810-4 PMC10236838 37269018

[CR24] Rodríguez OA, Matta NE (2001) Blood parasites in some birds from eastern plains of Colombia. Mem Inst Oswaldo Cruz 96. 10.1590/S0074-02762001000800026 10.1590/s0074-02762001000800026 11784943

[CR25] Ronquist F, Teslenko M, Van Der Mark P et al (2012) Mrbayes 3.2: efficient Bayesian phylogenetic inference and model choice across a large model space. Syst Biol 61:539–542. 10.1093/sysbio/sys029 22357727 10.1093/sysbio/sys029 PMC3329765

[CR26] Scaglione FE, Cannizzo FT, Pregel P, et al (2016) Emoparassiti in cornacchie grigie (*Corvus corone cornix*) in Piemonte. Vet Ital 52:111–116. 10.12834/VetIt.110.307.2 10.12834/VetIt.110.307.2 27188825

[CR27] Schmid S, Fachet K, Dinkel A, et al (2017) Carrion crows (*Corvus corone*) of southwest Germany: important hosts for haemosporidian parasites. Malar J 16. 10.1186/s12936-017-2023-5 10.1186/s12936-017-2023-5 PMC5596962 28899382

[CR28] Shurulinkov P, Spasov L, Stoyanov G, Chakarov N (2018) Blood parasite infections in a wild population of ravens (*Corvus corax*) in Bulgaria. Malar J 17. 10.1186/s12936-018-2179-7 10.1186/s12936-018-2179-7 PMC5771030 29338711

[CR29] Strehmann F, Becker M, Lindner K, et al (2023) Half of a forest bird community infected with haemosporidian parasites. Front Ecol Evol 11. 10.3389/fevo.2023.1107736

[CR30] Synek P, Munclinger P, Albrecht T, Votýpka J (2013) Avian haemosporidians in haematophagous insects in the Czech Republic. Parasitol Res 112:839–845. 10.1007/s00436-012-3204-3 23224608 10.1007/s00436-012-3204-3

[CR31] Valkiūnas G, Iezhova TA (2023) Insights into the biology of *Leucocytozoon* species (Haemosporida, Leucocytozoidae): why is there slow research progress on agents of leucocytozoonosis? Microorganisms 11(5):1251. 10.3390/microorganisms11051251 37317225 10.3390/microorganisms11051251 PMC10221462

[CR32] Valkiunas G (2004) Avian malaria parasites and other haemosporidia. (1st ed.). CRC Press. 10.1201/9780203643792

[CR33] Votýpka J, Lukeš J (2004) Oborník M (2004) Phylogenetic relationship of Trypanosoma corvi with other avian trypanosomes. Acta Protozool 43:225–231

[CR34] Yoshimura A, Koketsu M, Bando H, et al (2014) Phylogenetic comparison of avian Haemosporidian parasites from resident and migratory birds in Northern Japan. J Wildl Dis 50. 10.7589/2013-03-071 10.7589/2013-03-071 24484482

[CR35] Zélé F, Vézilier J, L’Ambert G, et al (2014) Dynamics of prevalence and diversity of avian malaria infections in wild *Culex pipiens* mosquitoes: the effects of *Wolbachia*, filarial nematodes and insecticide resistance. Parasit Vectors 7. 10.1186/1756-3305-7-437 10.1186/1756-3305-7-437 PMC4261254 25228147

[CR36] Zídková L, Cepicka I, Szabová J, Svobodová M (2012) Biodiversity of avian trypanosomes. Infect Genet Evol 12:102–112. 10.1016/j.meegid.2011.10.022 22080850 10.1016/j.meegid.2011.10.022

